# Pulmonary vein isolation without left atrial mapping

**Published:** 2007-08-01

**Authors:** Attila Kardos, Csaba Foldesi, Karoly Ladunga, Attila Toth, Tamas Szili-Torok

**Affiliations:** 1 Gottsegen Gyorgy Hungarian Institute of Cardiology; 2 Semmelweis University, Department of Radiology

**Keywords:** Pulmonary vein isolation, electroanatomical mapping, multislice computer tomography, image fusion

## Abstract

**Background:**

One of the crucial points during in most approaches developed for ablation of atrial fibrillation (AF) is the ability to identify the pulmonary vein (PVs) and to accurately locate their ostia. Objectives: The purpose of this case series was to investigate a simplified method for fusion of the multislice computer tomography (CT) derived 3D dataset with the electroanatomical map in order to facilitate the mapping procedure.

**Methods:**

In 5 consecutive patients (4 male) referred for catheter ablation of symptomatic drug-refractory paroxysmal atrial fibrillation contrast enhanced computer tomography was performed before the procedure and imported into an electroanatomical mapping system (Carto XP) using CartoMerge Image Integration Module. During the procedure a multipolar mapping catheter (Quick Star DS, Biosense Webster, Diamond Bar, CA, USA) was introduced to the coronary sinus (CS) to align the CSCT shell to the proper position. The CS potentials provided information to identify the ostium of the CS to achieve a more accurate fusion of the images. No mapping points were taken in the left atrium. The feasibility of the method was characterized by the distance of mapping points. Mapping, registration and outcome data were compared with a cohort of patients undergoing MRI image integration.

**Result:**

The mean distance between the mapping points taken in the CS by the Quick Star catheter and the CS CT surface was suitable (mean±SD, 1.4±0.3 mm). Full electrical isolation of the pulmonary veins could be achieved in all patients. The mean procedure and fluoroscopy time were 39 ± 22 and 134 ±38 min respectively, significantly decreased as compared to the MRI cohort.

**Conclusions:**

Highly accurate CT image and the electroanatomical map (EAM) fusion can be obtained by the Carto 3D electromanatomical mapping system using CS as the key anatomical structure for registration. Using this technique the mapping time of the left atrium can be reduced.

## Introduction

Pulmonary vein isolation is an alternative therapeutic option for patients with atrial fibrillation [1]. Although the outcome of patients undergoing different type of procedures shows progressive improvement, most of these methods remain rather complex, lengthy and carry a risk for even potentially lethal complications[2]. Improved imaging has the ability to diminish complication rate and improve outcome [3-5]. On the other hand extensive mapping procedure leads to an even longer procedure and fluoroscopy times. One of the new achievements in current imaging is the possible fusion between three dimensional off line CT or MRI images and electroanatomical maps. This reportedly increases procedural success [6,7]. The potential advantages and pitfalls of current merge technology in the catheter based treatment of atrial fibrillation are well described. Especially image integration of the electroanatomic map with the CT/MR image needs structures' relative stability of motions (distal pulmonary veins, aorta) to achieve minimal registration error [5]. The aim of the present study was to test a fundamentally different approach - i.e.  coronary sinus registration - during image integration to simplify the merging process. In order to demonstrate its feasibility we present a case series of 5 patients undergoing image fusion using our new method.

## Methods

### Image integration

**Figure 1:**
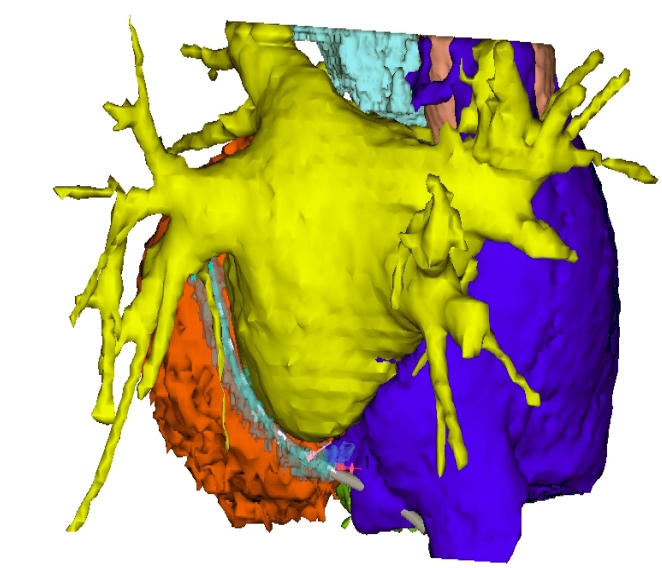
Cardiac CT derived endocardial shell of the heart reconstructed by Carto in postero-anterior view. Note the mapping catheter in the coronary sinus.

After Institutional Review Board approval in five patients (mean age: 55±8 years) referred to our institute for catheter ablation of symptomatic drug-refractory paroxysmal atrial fibrillation, General Electric UltraSpeed 8-slice imaging instrument was used for contrast enhanced computer tomography (CT) before the procedure. Following administration of 100 ml non-ionic iodinated contrast material by a single barrel injector (Medrad) at flow rate of 4 ml/sec ECG-gated, low-pitch helical scan (SnapShot) was performed in the feet-head direction resulted in thin axial slices reconstructed at a specific delay. The CT images were imported into an electroanatomical mapping system (Carto XP, Biosense Webster, Inc., Diamond Bar, CA, USA) using Carto Merge Image Integration Module in the electrophysiologic workstation. The left and right atrium (LA, RA) and coronary sinus (CS) were segmented from other cardiac structures and their endocardial shell was extracted for image registration. In the electrophysiology lab the right internal jugular vein was cannulated, and the mapping catheter (Quick Star DS, Biosense Webster, Inc., Diamond Bar, CA, USA) was introduced to the CS via the right internal jugular approach before trans-septal puncture to fit the CS CT shell. The CS potentials provided information to identify the ostium of the CS to achieve a more precise fusion of the images. Mean distance between the mapping points taken in the CS by the Quick Star catheter and the CSCT surface was measured. Then the catheter was introduced into the inferior and superior vena cava for registration and after the trans-septal puncture through femoral venous access into the pulmonary veins (PVs) to check the accuracy (alignment) of the image registration. No any other mapping points were taken in the left atrium. During radiofrequency ablations to isolate the PVs the ablation points were taken to achieve further improvement of image integration (Figure 1).

### Radiofrequency catheter ablation

**Figure 2:**
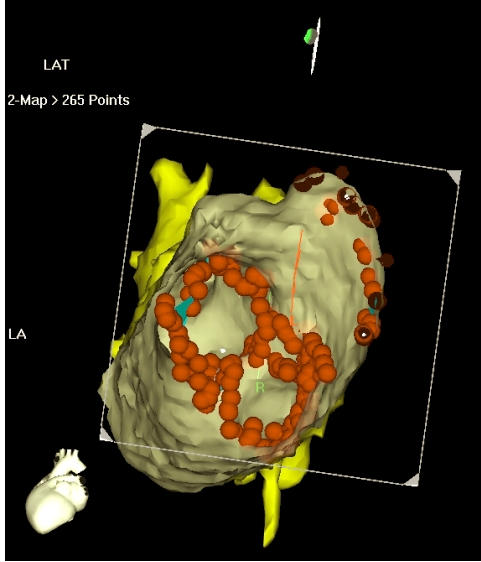
Endoluminal view of the left sided pulmonary veins after successful complete electrical isolation. The red dots demonstrate the radiofrequency lesions.

Initially after two successful transseptal puncture a circular mapping catheter (Lasso, Biosense Webster, Diamond Bar, CA, USA) was placed into one of the pulmonary veins. Circumferential ablation lines were created using a 3.5 mm irrigation tip catheter (Navistar Thermocool, Biosense Webster, Diamond Bar, CA, USA ). After complete isolation of the pulmonary veins, further lines were undertaken based on the history of the patients. In persistent and permanent atrial fibrillation, mitral annulus, appendage isolation, roof line, posteroinferior transversal line was created (Figure 2).

### Statistical analysis

Data are reported as mean ± standard deviation.  Procedural acute and follow up outcome data were compared with a cohort of 5 patients, where the image fusion was based on MRI images and was performed using full left atrial anatomical mapping using two-tailed Student t-test.

## Results

The CS ostium could be cannulated in each patients with the multipolar mapping catheter. The average registration procedure was with minimum use of fluoroscopy was 5 minutes.  Mean distance between the mapping points taken in the CS by the Quick Star catheter and the CS CT surface was 1.4 ±0.3 mm. The mean procedure time was 134±38 min.  The mean fluoroscopy time was 39±22 min. All pulmonary veins could be electrically isolated in all patients (100%). 4 out of 5 patients had no recurrences during the mean follow up of 8 months (ranging  6-14). Two patients had early appearances of left atrial tachycardias. In one patient these arrhythmias disappeared spontaneously, while in another patient propafenone therapy was initiated, and the patient responded to this favorably. Outcome data comparing ablation result of the two groups are summarized in Table 1.

## Discussion

The major finding of this study is that a simplified method for accurate fusion of MSCT images with electroanatomical mapping systems is feasible and indeed facilitates ablation procedures. Although the method requires further evaluation in a larger number of series it is a promising alternative to extensive and meticulous mapping of the left atrium in order to achieve acceptable fusion.

## The advantages of MSCT integration

Accurate catheter position and discrimination between scar tissue and poor contact may facilitate ablation procedures in patients with dilated atria and complex anatomy. Image integration is a tool which potentially can fulfill the above mentioned requirements during ablations. Although prior publications have demonstrated the feasibility of image integration during PV isolation, these attempts were based on anatomical mapping utilizing a roving mapping catheter in the left atrium [5,7]. Indeed, this results in an accurate fusion (i.e. registration), but lengthens the procedures significantly, in a situation where one of the major goal of further developments to diminish it. Our early experience shows that CT derived images can be precisely fused with the 3D electroanatomical map using the Carto 3D electroanatomical mapping system utilizing CS as the key anatomical structure for the registration process. The mapping time for pulmonary vein isolation can be dramatically reduced by this technique, as compared to the MR cohort. This seems to be a major advantage especially in patients where extensive ablation procedure is planned.

### Alternative possibilities

This approach opens perspective for other type of arrhythmias as well. The same method can easily be applied for the mapping of right atrial tachycardias. During ablation of ventricular tachycardias using the retrograde transaortic route even the aorta can itself be used for facilitating the fusion. Our experience suggest, that indeed blood vessels visualized by the means of CT, can be used when the vessels has three dimensional configuration.

### Limitations of the study

Although this initial experience is rather promising it seems however, results should be with caution since this was a small series study. The other main limitation of this study is that it was not randomized. However compared with a similar number of cohort using MRI registration (where coronary sinus was not available), the fluoroscopy and procedure times were significantly shorter. Finally, we do not have large number of patients to compare clinical outcome in order to be sure that shorter mapping will not be counteracted by the worsening of clinical outcome.

In conclusion, the major finding of this study is that the method we developed seems to be technically feasible and our data suggest that indeed this shortens procedures and decreases fluoroscopy time.

## Figures and Tables

**Table 1:**
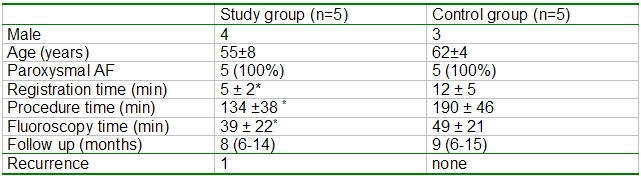


*P<0.05
